# Convergence of Humans, Bats, Trees, and Culture in Nipah Virus Transmission, Bangladesh

**DOI:** 10.3201/eid2309.161922

**Published:** 2017-09

**Authors:** Emily S. Gurley, Sonia T. Hegde, Kamal Hossain, Hossain M.S. Sazzad, M. Jahangir Hossain, Mahmudur Rahman, M.A. Yushuf Sharker, Henrik Salje, M. Saiful Islam, Jonathan H. Epstein, Salah U. Khan, A. Marm Kilpatrick, Peter Daszak, Stephen P. Luby

**Affiliations:** icddr,b, Dhaka, Bangladesh (E.S. Gurley, S.T. Hegde, K. Hossain, H.M.S. Sazzad, M.J. Hossain, M.A. Yushuf Sharker, M.S. Islam, S.U. Khan, S.P. Luby);; Centers for Disease Control and Prevention, Atlanta, Georgia, USA (S.T. Hegde, S.P. Luby);; Ministry of Health and Family Welfare, Bangladesh, Dhaka (M. Rahman);; Johns Hopkins Bloomberg School of Public Health, Baltimore, Maryland, USA (H. Salje);; Institut Pasteur, Paris, France (H. Salje); EcoHealth Alliance, New York, New York, USA (J.H. Epstein, P. Daszak);; University of Florida, Gainesville, Florida, USA (S.U. Khan);; University of California, Santa Cruz, California, USA (A.M. Kilpatrick);; Stanford University, Stanford, California, USA (S.P. Luby)

**Keywords:** Nipah virus, NiV, viruses, infections, case–control study, human behavior, humans, bats, trees, culture, virus transmission, epidemiology, convergence, date palm sap, vector-borne infections, zoonoses, Bangladesh

## Abstract

Preventing emergence of new zoonotic viruses depends on understanding determinants for human risk. Nipah virus (NiV) is a lethal zoonotic pathogen that has spilled over from bats into human populations, with limited person-to-person transmission. We examined ecologic and human behavioral drivers of geographic variation for risk of NiV infection in Bangladesh. We visited 60 villages during 2011–2013 where cases of infection with NiV were identified and 147 control villages. We compared case villages with control villages for most likely drivers for risk of infection, including number of bats, persons, and date palm sap trees, and human date palm sap consumption behavior. Case villages were similar to control villages in many ways, including number of bats, persons, and date palm sap trees, but had a higher proportion of households in which someone drank sap. Reducing human consumption of sap could reduce virus transmission and risk for emergence of a more highly transmissible NiV strain.

Emerging zoonoses pose a substantial threat to human health and well-being (*1*). Some of the most devastating human disease pandemics have been caused by diseases originating in livestock or wildlife, including HIV infection, influenza, bubonic plague, and a large Ebola outbreak in West Africa (*1*,*2*). For this reason, there is considerable scientific and public health interest in predicting which emerging pathogens have the potential to cause pandemics so that these pandemics can be prevented. Emerging lethal zoonotic pathogens that have crossed the species barrier and can be transmitted from 1 person to another, albeit without sustained person-to-person transmission, are particularly concerning because they could evolve to become more highly transmissible and cause large outbreaks or pandemics (*3*). It is therefore critical to focus resources on limiting the opportunity of these pathogens to spillover from wildlife and livestock to infect persons and to better adapt to human hosts.

Effectively preventing cross-species transmission of zoonotic pathogens depends on our ability to determine how transmission occurs, including transmission pathways and determinants of human risk. Efforts to identify and predict risky geographic areas for emerging zoonoses have focused primarily on publicly available data, remote sensing of species habitat, and other large-scale population measures (*4*,*5*). A major limitation of these risk mapping approaches is that they typically rely on crude measures of spatial risk, including presence or absence of species or population densities. Human behavior patterns are rarely taken into account, although the risk for transmission probably involves complex, time-varying interactions between humans and their environment that are often driven by culture, climate, and economic development (*1*,*6*,*7*).

Infection with Nipah virus (NiV), an emerging zoonotic pathogen, can cause encephalitis in humans; the virus can also be transmitted between humans, although somewhat inefficiently (*8*). NiV was first identified as the etiologic agent causing outbreaks in pigs and encephalitis in humans in Malaysia and Singapore during 1998–1999 (*9*). Shortly thereafter, this virus was identified as the cause of outbreaks of human encephalitis in Bangladesh and India during 2001 (*10*,*11*). Nearly every year since 2001, NiV has caused outbreaks among humans in Bangladesh; cases are also reported in bordering areas of India (*12*). Initial spillovers during these outbreaks have been amplified by person-to-person transmission; the largest of these outbreaks involved 66 persons, primarily patients and healthcare workers, in Siliguri, India, in 2001 (*10*). In addition, an outbreak in Faridpur, Bangladesh, in 2004 involved 5 generations of transmission (*13*). Although the case-fatality rate for patients in Malaysia and Singapore was ≈40%, it exceeds 70% in Bangladesh and India (*12*).

The natural reservoir for NiV is Old World fruit bats of the genus *Pteropus*, which are found in eastern Africa and throughout Asia, Australia, and the Pacific islands (*14*,*15*). Antibodies against NiV or NiV-like viruses have been found in pteropid bats throughout Asia, including Malaysia, Thailand, Cambodia, India, and Bangladesh (*16*–*20*). *Pteropus medius* (formerly *P. giganteus*) is the only pteropid bat species present in India and Bangladesh and is the putative reservoir for NiV in this region (*21*). The widespread evidence of henipavirus infection in *Pteropus* bats suggests that this virus may have co-evolved with bats and has probably been present in these areas for as long as the bats have been there. Infected bats shed NiV in their saliva and urine (*22*,*23*), and spillover might occur between humans and bats throughout this region. Types of contact that could result in NiV transmission include hunting bats for human consumption; living nearby and under bat roosts; and sharing food resources, including bats drinking date palm sap and humans consuming fruit partially eaten by bats (*8*,*16*,*24*). Despite this information, the geographic scope and scale of reported cases of infection with NiV remains limited: only Bangladesh regularly reports cases (*25*). Furthermore, even within Bangladesh, there is unexplained substantial spatial heterogeneity in case occurrence; virtually all cases are detected in the central and northwestern parts of the country (Figure 1) (*25*).

**Figure 1:**
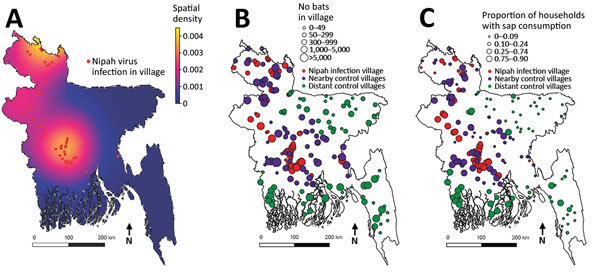
A) Locations of identified bat-to-human transmission of Nipah virus and spatial intensity of transmission events, Bangladesh, 2001–2012. B) Relative sizes of the *Pteropus medius* bat populations in case and control villages (including within 5 km of each village). C) Proportion of households in case and control villages with persons who regularly consume fresh date palm sap.

The purpose of our study was to identify differences in endogenous risk and risky human behavior across these areas that drive these human disease patterns. Outbreak investigations in Bangladesh showed that a major risk factor for NiV infection was consumption of raw date palm sap, a national delicacy (*12*). Date palm trees (*Phoenix sylvestris*) are tapped overnight to collect the sap in clay pots, and the sweet sap is retrieved from the tree first thing in the morning and drunk raw (*26*). Wildlife studies have shown that date palm sap is commonly consumed by *Pteropus* bats, particularly during winter months when other fruits are not available (*27*). We sought to understand causes of geographic variation in NiV transmission from bats to humans across Bangladesh. We performed a large-scale, case–control study that used villages as study units and quantified the distribution of bats, humans, date palm sap trees, and human behavior that might influence interactions with bats, such as date palm sap harvesting and consumption.

## Methods

### Data Collection

We conducted a village-level case–control study to identify characteristics associated with NiV transmission from bats to humans in Bangladesh. Case villages were those in which human infections were identified during 2001–2012 with no evidence that the source of infection was another human case of infection with NiV and thus was probably caused by spillover from bats. We mapped these case villages and drew 50-km buffers around them to define the area of Bangladesh to which NiV was endemic (Figure 1, panel B). This distance chosen was arbitrary but reasonably represents the distance that a person could travel within a day, even without access to good roads, and is within the typical nightly foraging radius of *Pteropus* bats, which has been observed as 20–50 km (*28*–*30*). We then selected control villages by randomly generating 2 sets of geographic points on a map. The first set was chosen from within the area to which NiV was endemic (nearby controls) but >5 km from a case village. The second set was chosen from points >50 km from case villages (distant controls). We chose control villages near and distant from villages with cases of infection with NiV to determine if characteristics driving transmission were different at varying spatial scales. We sought to enroll 75 controls from nearby villages and 75 controls from distant villages.

Our approach for identifying control villages could have misclassified some villages where cases of infection with NiV have occurred but gone undetected. However, all outbreaks due to NiV have been observed exclusively in the part of the country that includes our case and nearby control villages, despite the fact that surveillance for outbreaks frequently identifies outbreaks of other diseases throughout the country (*31*).

We collected data for study villages at 2 time points: during December 2011−February 2012 and during December 2012−February 2013. These times were chosen to align our surveys with the season that has a high incidence NiV infection in Bangladesh (*12*). Trained data collection teams used hand-held devices to identify the latitude and longitude of the randomly selected points and enrolled the village with that coordinate or the village located closest to that point as a control village. Teams visited case and control villages and identified a group of key village informants who assisted with mapping the village boundary and estimating the number of households located in each village. The teams then asked local residents to identify all of the bat roosts they knew of in their village and within 5 km of the village boundary. Trained data collectors located all of the roosts and counted the number of *Pteropus* bats roosting. In addition, they counted all date palm sap trees in the village and within 500 m of the village boundary.

We then requested that 25 randomly selected households from each village participate in a structured survey. After identifying the village boundary, the field team used a random number table to choose a cardinal direction: north, east, south, or west. The household on the edge of the village in that direction was approached for participation. The team then divided the estimated number of households in the village by the desired sample size (25) and skipped that number of households to choose the next for participation. This process continued until 25 households were enrolled. For villages with <25 households, all households were enrolled. Data collectors administered the structured survey to an adult household member to obtain data for household demographics, date palm sap consumption practices, experience with observing and hunting bats, number of fruiting trees on their household premises, and behavior regarding eating fruit with animal bite marks off the ground.

### Data Analysis

To estimate the number of persons in each village, we multiplied the number of estimated households in each village by the mean number of household residents in households sampled for the study from that particular village. We measured data for each village by direct observation, such as the number of bats roosting, or through the surveys at sampled households. We compared case villages to both sets of controls in terms of human and bat population size, as well as human behavior patterns regarding date palm sap and fruit consumption.

We estimated means and proportions with 95% CIs and compared case and control villages by using generalized linear models with binomial distributions and a logit link, which used robust variance estimates to account for model misspecification. For exposures measured at the household level, we accounted for clustering within the village in the model. Analyses were conducted by using the generalized linear model package in Stata version 13.0 (StataCorp LLC, College Station, TX, USA). Variables that were highly skewed were log-transformed to equalize leverage.

We used multivariable logistic regression models to estimate independent associations (odds ratios with 95% CIs) between village characteristics and being a village with a case of NiV infection. For multivariable regression, each village had 1 value for each exposure, and household-level data from each village was aggregated to estimate the proportion of all village households reporting each exposure or behavior. Therefore, we did not need to account for clustering of observations, but we did calculate the 95% CIs for the odds ratios by using robust variance to account for imprecision in exposures estimated from a sample of households. We first built an inclusive model comparing each set of controls to the case villages on the basis of our a priori hypothesis that NiV spillover risk is determined by human population, number of bats present, number of date palm trees, and proportion of persons in the village who commonly consumed raw date palm sap. In addition, any other behavior patterns that were associated with an increased risk for NiV spillover by univariable analysis were also included in the model. Associations were considered statistically significant if p values were <0.05.

### Ethical Considerations

All study participants provided informed consent before participation. The study protocol was reviewed and approved by the institutional review board of the icddr,b.

## Results

Data collection teams visited all 60 case villages and 73 nearby control villages and 74 distant control villages where they surveyed 5,024 persons (Figure 1, panel B). Three selected control villages could not be visited because of local security concerns or logistical constraints.

**Figure 2:**
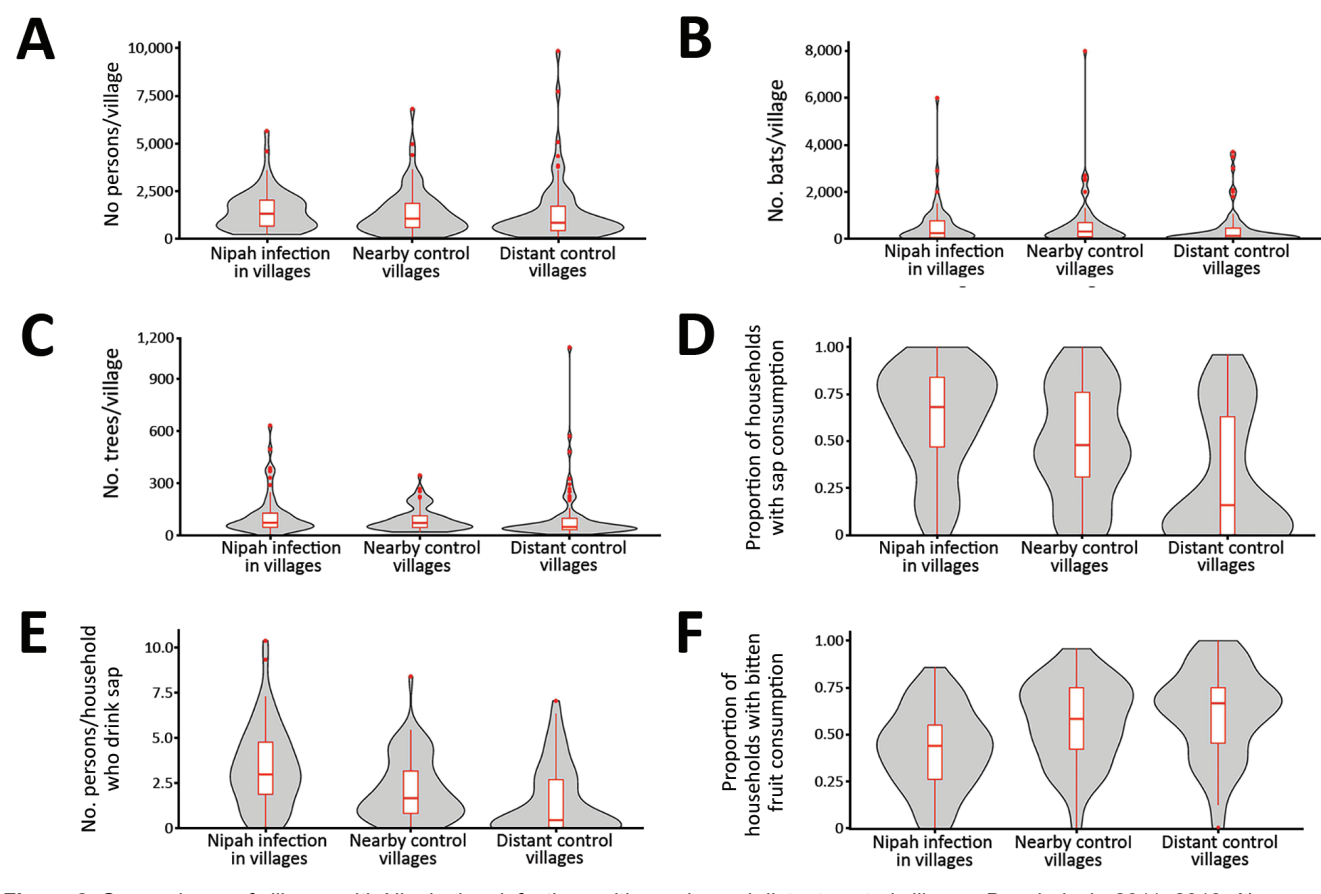
Comparisons of villages with Nipah virus infections with nearby and distant control villages, Bangladesh, 2011–2013. A) Human population size, B) *Pteropus medius* bat population size, C) no. date palm trees, D) proportion of households with members who consume fresh date palm sap, E) average no. of persons per household who consume fresh date palm sap, and F) proportion of households that reported their residents eat bitten fruits dropped on the ground. Gray shading in violin plots indicates distribution of values for each variable. Box plots indicate 25th and 75th percentiles (bottom and top lines), medians (horizontal lines within boxes), and 95 CIs (whiskers). Red dots indicate maximum (outlier) values.

Villages that had cases of infection with NiV were similar to nearby and distant control villages for most characteristics, including human population, bat population, and number of date palm sap trees (Table 1; Figure 2). However, both groups of control villages had a lower proportion of households who reported that >1 person commonly drank fresh date palm sap than households in case villages (61% in case villages vs. 49% in nearby control villages and 31% in distant control villages) (Table 1; Figure 1, panel C; Figure 2).

**Table 1:** Characteristics of villages with cases of Nipah virus infection and control villages, Bangladesh 2011–2013*

Characteristic	Villages with cases, n = 60	Nearby control villages, n = 73	p value†	Distant control villages, n = 74	p value‡
Human population					
No. persons in village	1,476 (1,202–1,749)	1.389 (1,102–1,676)	0.20	1,392 (1,010–1,774)	0.10
No. persons/km^2^	1,168 (1,167–2,169)	1,173 (592–1,754)	0.78	1,335 (456–2,213)	0.95
*Pteropus* bat population					
Proportion of villages where *P. medius* bats were observed roosting or within 5 km of village boundary	0.85 (0.76–0.94)	0.86 (0.78–0.94)	0.86	0.76 (0.66–0.86)	0.19
No. bats roosting in village or within 5 km of village boundary	554 (319–789)	620 (364–875)	0.60	407 (226–587)	0.37
Proportion of respondents reporting large fruit bats					
Roosted nearby during the day in past month	0.25 (0.17–0.34)	0.37 (0.29–0.45)	0.060	0.40 (0.31–0.49)	0.024
Fly overhead at dusk	0.51 (0.43–059)	0.64 (0.56–0.70)	0.019	0.77 (0.71–0.83)	<0.001
Visit fruit trees at night	0.43 (0.35–0.51)	0.52 (0.45–0.60)	0.10	0.53 (0.45–0.61)	0.090
Date palm sap and fruiting trees					
No. trees in village within a 500-m radius of village boundary	120 (88–152)	95 (78–111)	0.91	101 (65–138)	0.14
Proportion of households with fruiting trees on premises	0.97 (0.94–0.99)	0.97 (0.94–0.98)	0.81	0.94 (0.92–0.96)	0.14
No. fruiting trees on each household premise	56 (46–68)	52 (43–61)	0.81	108 (45–170)	0.47
Human behavior					
Proportion of villages with >1 date palm sap collector	0.60 (0.47–0.63)	0.40 (0.29–0.52)	0.026	0.51 (0.40–0.63)	0.32
No. sap collectors in villages	4.5 (1.8–7.3)	2.3 (1.0–3.6)	0.41	3.7 (1.9–5.6)	0.54
Proportion of villages with >1 fresh date palm sap seller	0.38 (0.28–0.51)	0.32 (0.21–0.43)	0.45	0.39 (0.26–0.51)	0.92
No. (%) fresh sap sellers in villages	1.9 (0.6)	0.9 (0.2)	0.16	2.4 (0.6)	0.47
Proportion of households where >1 person drank raw sap	0.61 (0.54–0.68)	0.49 (0.42–0.56)	0.014	0.31 (0.24–0.39)	<0.001
Proportion of households where someone drank raw sap >1×/wk during the past harvest season	0.35 (0.27–0.43)	0.29 (0.23–0.35)	0.26	0.21 (0.16–0.27)	0.005
No. household residents who drank >1 glass of raw date palm sap when in season	3.3 (2.7–3.9)	2.1 (1.8–2.5)	0.001	1.5 (1.1–1.9)	<0.001
Proportion of villages where >1 household fed raw date palm sap to livestock	0.16 (0.10–0.21)	0.12 (0.06–0.18)	0.66	0.14 (0.08–0.21)	0.78
Proportion of villages where >1 person hunted bats	0.53 (0.40–0.66)	0.64 (0.53–0.75)	0.22	0.27 (0.17–0.38)	0.002
Proportion of households that reported residents ate bitten fruits dropped on the ground	0.42 (0.37–0.48)	0.58 (0.53–0.62)	<0.001	0.66 (0.61–0.71)	<0.001

*Values are mean (95% CI) except as indicated. †Comparison of villages with cases of Nipah virus infections with nearby control villages by using generalized linear models that account for correlations within villages for characteristics measured in household surveys. ‡Comparison of villages with cases of Nipah virus infections with distant control villages by using generalized linear models that account for correlations within villages for characteristics measured in household surveys.

In addition, the average number of household residents who drank >1 glass of raw sap when it was in season was higher in case villages (3.3 glasses) than in nearby control villages (2.1 glasses) and distant control villages (1.5) (Table 1; Figure 2, panel E). A larger proportion of villages that had cases of infection with NiV had >1 bat hunter than distant control villages (53% vs. 27%; p = 0.002) (Table 1). Households in nearby and distant control villages were more likely to report that someone in the house ate fruits bitten by animals off the ground and saw bats roosting nearby during the day and flying overhead at dusk than were households in villages with cases of infection with NiV (Table 1).

Multivariable analyses showed that, compared with nearby control villages, each additional 10% increase in the proportion of households reporting that someone regularly consumed raw sap was associated with a 6.39 (95% CI 1.61–25.40) increase in odds of being a village with cases of NiV infection (Table 2). Compared with distant control villages, the odds of being a village with cases of NiV infection were 1.18 (95% CI 1.02–1.37) times higher for each order of magnitude increase in bat populations and 26.97 (95% CI 5.98–121.67) times higher for each 10% increase in households that reported someone who regularly consumed sap.

**Table 2:** Odds ratios from logistic regression models estimating associations between village characteristics and Nipah virus spillovers, Bangladesh, 2011–2013*

Characteristic	OR (95% CI) for villages with NiV infections vs. nearby control villages	p value	OR (95% CI) for villages with NiV infections vs. distant control villages	p value
Per each order of magnitude increase in no. persons in village	1.36 (0.90–2.07)	0.14	1.57 (0.90–2.6)	0.12
Per each order of magnitude increase in no. bats <5 km from village	1.00 (0.86–1.16)	0.97	1.18 (1.02–1.37)	0.029
Per each order of magnitude increase in no. date palm sap trees <5 km from village	0.75 (0.49–1.12)	0.16	.69 (0.45–1.04)	0.078
Per each 10% increase in households reporting that someone consumed raw date palm sap during the harvest season	6.39 (1.61–25.40)	0.008	26.97 (5.98–121.67)	<0.001
Per each 1% increase in villages reporting that someone hunts bats	NA	NA	1.80 (0.80–4.06)	0.16

*CIs were calculated by using robust variance. NA, not applicable; NiV, Nipah virus; OR, odds ratio.

## Discussion

NiV is a highly fatal pathogen and poses a risk for pandemic spread because it can be transmitted from person-to-person. To reduce opportunities for a more transmissible strain to emerge, which could lead to regional outbreaks or a pandemic, we must prevent spillover from bats to humans. Bangladesh is the only place where spillover events are predictably identified each year. Therefore, preventing bat-to-human transmission of NiV in rural Bangladesh should be a global public health priority.

Our study reported 2 key findings to achieve this priority. First, our data suggest that human infection, and as a result, selective pressure to adapt to humans (*3*), is determined by the joint probability of a human consuming raw sap in rural Bangladesh and of sap being contaminated by the urine or saliva of a bat that is shedding the virus (*22*,*32*). Bangladesh has a population of 160 million persons, and according to a United Nations report (http://esa.un.org/unpd/wup/Country-Profiles/), 70% of Bangladeshi residents in 2015 lived in rural areas where date palm sap is collected. We found that >30% of rural households have >l regular date palm sap drinker, which implies that there are millions of persons drinking fresh sap each year. The reservoir for NiV (the *P. medius* bats) is nearly ubiquitous across the landscape (Figure 1, panel B), and previous studies show that bat visits to date palm sap trees are common (*27*), which suggests that much of the fresh date palm sap consumed is probably contaminated with bat saliva or excreta. Despite this finding, human infections with NiV are rare, which suggests that shedding of transmissible virus by bats is also rare during the date palm sap harvesting season or occurs too infrequently to cause human infection.

Date palm sap consumption was common in control villages, although less common than in case villages. These findings suggest that we need not eliminate date palm sap consumption to reduce NiV spillovers. Date palm sap is deeply rooted in Bengali culture (*32*), and because the risk associated with consumption at the individual level is low, eliminating this practice could be difficult. However, even if we are unable to eliminate sap consumption, modest reductions in consumption of contaminated date palm sap could meaningfully reduce incidence rates for infection with NiV. Case villages were also more likely to have more bats roosting nearby than distant control villages but not nearby control villages. This finding suggests that although large increases in bat population sizes could increase risk for spillover, odds ratios indicate that human behavior patterns are a greater risk for driving NiV transmission than bat population size. Extermination of bats would not be an appropriate approach to mitigating the risk for NiV infection because of the major ecologic role of *P. medius* bats in tree pollination and seed dispersal. Public health messages during outbreaks stress the need for bats in the local ecology, but greater efforts to preserve bat habitat during outbreaks should be considered.

Second, our data suggest that we should target interventions to communities that consume large amounts of raw sap. We identified that consumption of date palm sap was common in many areas across Bangladesh, even in areas where no cases of infection with NiV have been detected. The ability to target resources is key when funding for public health prevention is limited. Our data suggest that areas with high consumption of raw sap should be targeted for enhanced surveillance to track changes in NiV epidemiology and quickly respond to outbreaks and for interventions to interrupt transmission through consumption of contaminated sap. Interventions to reduce human consumption of contaminated sap have been developed and include efforts to reduce fresh sap consumption in general and using physical barriers to keep bats from accessing and contaminating sap (*32*–*36*). However, sustained changes in behavior patterns regarding consumption of date palm sap will probably require long-term efforts to promote these interventions because this consumption is ingrained in local culture, and there is evidence that knowledge per se about risk for infection with NiV is not associated with behavior patterns regarding date palm sap consumption in areas to which NiV is endemic (*32*,*36*).

Residents of nearby and distant control villages were more likely to report seeing bats roosting nearby during the day. One possible explanation for this finding could be an association between experiencing an outbreak of infection with NiV and destruction of bat habitat. Local investigation teams have observed that residents in villages in which outbreaks have occurred often cut down trees in which bats roost within the village after the outbreak. Residents of nearby and distant control villages were also more likely than residents of case villages to report eating animal-bitten fruits off the ground. Investigations of the first outbreak of infections with NiV in Malaysia suggested that the most probable pathway of transmission from bats to pigs was through consumption of bat-bitten fruits (*37*). However, there is no evidence that this transmission route plays a major role in human infections in Bangladesh, despite more than a decade of investigation (*38*), and this study provides further evidence that this factor is not a major contributor to human infection in this setting, given the strong association between human date palm sap consumption and being a village with cases of NiV infection. No outbreaks of infection with NiV have been linked with bat hunting, but Old World fruit bats are hypothesized to be reservoir hosts for several major zoonotic pathogens, including Marburg virus and Ebola virus (*39**,**40*). In addition to NiV, our group reported evidence of 55 novel viruses in *P. medius* bats (*41*), and evidence for human exposure to these or other bat-borne pathogens through this type of bat contact should be explored. Efforts to reduce bat hunting would be beneficial for conservation of these species and reduction of disease risk.

Our study objective was to identify the major drivers of spatial patterns of NiV spillovers across Bangladesh by drawing upon evidence we have about individual risk factors for NiV infection. There might be other rare drivers of risk that were not detected because of limited statistical power. However, these drivers would have a smaller role in explaining disease risk than those identified in this study. *P. medius* bats are found throughout Bangladesh (*42*) but spillover of NiV to humans could be driven by spatial or temporal variation in NiV incidence in bats. More evidence about this possible contributor to spatial heterogeneity would improve our understanding of risk.

Our study provides an example of how epidemiologic studies can be used to describe the ecologic drivers of zoonotic disease emergence. The risk for cross-species transmission is complex and depends on the presence of reservoir hosts and permissive contact patterns with humans, as well as the frequency of these interactions. Future studies to explain spatial risk for similar emerging zoonotic infections should incorporate data on all aspects of the transmission, including human behavior patterns.
